# Sperm chromatin structure assay (SCSA^®^) and flow cytometry-assisted TUNEL assay provide a concordant assessment of sperm DNA fragmentation as a function of age in a large cohort of approximately 10,000 patients

**DOI:** 10.1186/s12610-023-00208-9

**Published:** 2023-11-30

**Authors:** Paria Behdarvandian, Ali Nasr-Esfahani, Marziyeh Tavalaee, Kosar Pashaei, Nushin Naderi, Zahra Darmishonnejad, Jorge Hallak, Robert J. Aitken, Parviz Gharagozloo, Joël R. Drevet, Mohammad Hossein Nasr-Esfahani

**Affiliations:** 1 Department of Animal Biotechnology, Reproductive Biomedicine Research Center, Royan Institute for Biotechnology, ACECR, Isfahan, 8165131378 Iran; 2 Isfahan Fertility and Infertility Center, Isfahan, Iran; 3 Androscience, Science and Innovation Center in Andrology and High-Complex Clinical and Research Andrology Laboratory, Sao Paulo, 04534-011 Brazil; 4 Priority Research Centre for Reproductive Science, Discipline of Biological Sciences, School of Environmental and Life Sciences, College of Engineering Science and Environment, University of Newcastle, Callaghan, NSW 2308 Australia; 5 CellOxess LLC, Ewing, NJ 08628 USA; 6 Faculty of Medicine, Université Clermont Auvergne, GReD Institute, CRBC, 63000 Clermont-Ferrand, France

**Keywords:** Spermatozoa, Ageing, DNA fragmentation, Male fertility, Reproductive health, Spermatozoïdes, Vieillissement, Fragmentation de l'ADN, Fertilité masculine, Santé reproductive

## Abstract

**Background:**

Sperm DNA integrity is increasingly seen as a critical characteristic determining reproductive success, both in natural reproduction and in assisted reproductive technologies (ART). Despite this awareness, sperm DNA and nuclear integrity tests are still not part of routine examinations for either infertile men or fertile men wishing to assess their reproductive capacity. This is not due to the unavailability of DNA and sperm nuclear integrity tests. On the contrary, several relevant but distinct tests are available and have been used in many clinical trials, which has led to conflicting results and confusion. The reasons for this are mainly the lack of standardization between different clinics and between the tests themselves. In addition, the small number of samples analyzed in these trials has often weakened the value of the analyses performed. In the present work, we used a large cohort of semen samples, covering a wide age range, which were simultaneously evaluated for sperm DNA fragmentation (SDF) using two of the most frequently used SDF assays, namely the TUNEL assay and the sperm chromatin structure assay (SCSA®). At the same time, as standard seminal parameters (sperm motility, sperm morphology, sperm count) were available for these samples, correlations between age, SDF and conventional seminal parameters were analyzed.

**Results:**

We show that the SCSA® and TUNEL assessments of SDF produce concordant data. However, the SDF assessed by TUNEL is systematically lower than that assessed by SCSA®. Regardless of the test used, the SDF increases steadily during aging, while the HDS parameter (High DNA stainability assessed via SCSA®) remains unchanged. In the cohort analyzed, conventional sperm parameters do not seem to discriminate with aging. Only sperm volume and motility were significantly lower in the oldest age group analyzed [50–59 years of age].

**Conclusions:**

In the large cohort analyzed, SDF is an age-dependent parameter, increasing linearly with aging. The SCSA® assessment of SDF and the flow cytometry-assisted TUNEL assessment are well correlated, although TUNEL is less sensitive than SCSA®. This difference in sensitivity should be taken into account in the final assessment of the true level of fragmentation of the sperm nucleus of a given sample. The classical sperm parameters (motility, morphology, sperm count) do not change dramatically with age, making them inadequate to assess the fertility potential of an individual.

## Résumé

**Contexte:**

l'intégrité de l'ADN des spermatozoïdes est de plus en plus considérée comme une caractéristique essentielle déterminant le succès de la reproduction, tant dans la reproduction naturelle que dans les techniques de reproduction assistée (AMP). Malgré cette prise de conscience, les tests d'intégrité nucléaire des spermatozoïdes ne font toujours pas partie des examens de routine pour les hommes infertiles ou fertiles souhaitant évaluer leur capacité de reproduction. Cette situation n'est pas due à l'indisponibilité des tests. Au contraire, plusieurs tests pertinents mais distincts sont disponibles et ont été utilisés dans de nombreux essais cliniques, ce qui a donné lieu à des résultats contradictoires et à une certaine confusion. Les raisons en sont principalement le manque de normalisation entre les différentes cliniques et entre les tests eux-mêmes. En outre, le petit nombre d'échantillons analysés dans ces essais a souvent affaibli la valeur des analyses effectuées. Dans le présent travail, nous avons utilisé une vaste cohorte d'échantillons, couvrant une large tranche d'âge, évalués simultanément pour la fragmentation de l'ADN des spermatozoïdes à l'aide de deux des tests les plus fréquemment utilisés, à savoir le test TUNEL et le test de la structure de la chromatine des spermatozoïdes (SCSA®). Parallèlement, comme les paramètres séminaux standard (motilité, morphologie, numération) étaient disponibles pour ces échantillons, les corrélations entre l'âge, le niveau de fragmentation et les paramètres séminaux conventionnels ont été analysées.

**Résultats:**

Nous montrons que les évaluations SCSA® et TUNEL produisent des données concordantes. Cependant, le SDF évalué par TUNEL est systématiquement plus faible que celui évalué par SCSA®. Quel que soit le test utilisé, la fragmentation augmente régulièrement au cours du vieillissement, alors que le paramètre HDS (« High DNA stainability» évalué par le test SCSA®) reste inchangé. Dans la cohorte analysée, les paramètres spermatiques conventionnels ne semblent pas varier avec le vieillissement. Seuls le volume et la mobilité des spermatozoïdes étaient significativement plus faibles dans le groupe d'âge le plus élevé analysé [50–59 ans].

**Conclusions:**

Dans la grande cohorte analysée, la fragmentation de l'ADN spermatique est un paramètre dépendant de l'âge, augmentant linéairement avec le vieillissement. L'évaluation du SDF par SCSA® et l'évaluation via le test TUNEL assistée par cytométrie de flux sont bien corrélées, bien que le TUNEL soit moins sensible que le SCSA®. Cette différence de sensibilité doit être prise en compte dans l'évaluation finale du niveau réel de fragmentation du noyau des spermatozoïdes d'un échantillon donné. Les paramètres classiques du sperme (motilité, morphologie, nombre de spermatozoïdes) ne changent pas de façon spectaculaire avec l'âge, ce qui les rend inadéquats pour évaluer le potentiel de fertilité d'un individu.

## Introduction

Over the past three decades, an increase in the age of couples at the time of conception of the first child has been observed worldwide. This phenomenon has been attributed primarily to socioeconomic reasons, including increased life expectancy, later marriage, widespread use of contraception, increased cost of living, … which causes young couples to postpone their desire to conceive [1, 2]. Regardless of maternal age, some reports indicate that increasing male age is associated with decreased sperm quality, decreased fertility [3, 4], higher miscarriage rates [5] and susceptibility of offspring to conditions such as autism, bipolar disorder, achondroplasia, schizophrenia, … [4, 6–14].

Several mechanisms have been proposed to explain poor sperm quality with age: 1) decreased Sertoli and Leydig cell function leading to alterations in reproductive hormones and, consequently, poor spermatogenesis [15, 16]; 2) decreased seminal vesicle and prostate function associated with lower semen volume and reduced sperm motility [1, 10, 17]. Poor spermatogenesis leads to decreased gene expression [18], decreased DNA repair capacity, increased apoptosis, abnormal chromatin/chromosome structure [19], and altered epigenetic marks in differentiating germ cells, among others [20–22]. In addition, it must be taken into account that, unlike female germ cells, spermatogonial stem cells (SSCs) replicate continuously over the course of a man's life. This means that in a 25-year-old man, SSCs have undergone approximately 350 replication cycles, whereas in a 45-year-old man, this number rises to approximately 750 replication cycles [23], so it is clear that the risk of replication errors leading to de novo mutations is likely to be higher in older men than in younger men [24]. In couples with older male partners, this is thought to account for some of the infertility situations, as well as possible negative impacts on the next generation [2, 12, 13, 25].

It is commonly accepted that a major cause of increased sperm mutational load in aging men is oxidative in nature [2, 13, 26–28]. Oxidative damage to the sperm nucleus has been associated with nuclear alterations beginning with DNA base oxidation and extending up to chromatin decondensation and DNA fragmentation, which must be corrected by the oocyte machinery after fertilization [29–34]. Sperm DNA fragmentation (SDF) has been correlated with a higher rate of sperm chromosomal abnormalities [35–37], increased miscarriage rates and problems with foetal development and in the progeny [3, 38–45].

One study involving a large cohort (> 15,000 samples) showed that SDF, as measured by the sperm chromatin structure assay (SCSA®), was positively correlated with patient age [46]. A second study [47], also based on the assessment of SDF of just over 25,000 semen samples via the SCSA® test, also concluded that SDF was positively associated with age. However, some reports in which SDF was assessed using different tests have suggested that male age has no effect on SDF levels [48, 49]. It is unclear whether these discrepancies stem from the different tests used to assess SDF and/or in the cohorts analyzed in terms of size and characteristics. In an attempt to clarify this issue, we designed the present study on a large cohort (approximately 10,000 semen samples) in which SDF was analyzed concomitantly using the SCSA® and TUNEL assays, both performed by flow cytometry (FCM). The SDF data obtained with the two assays were then compared and correlated with the age of the patients as well as with standard semen parameters when available.

## Materials & methods

This study was approved by the Scientific Ethics Committee of the Royan Institute under the reference: IR.ACECR.ROYAN.REC.1401.031.

### Study samples

In this retrospective study, 10,000 semen samples from infertile couples referred to the Isfahan Fertility and Infertility Center (IFIC) between March 2018 and August 2022 were used.

### Semen analysis

All samples were evaluated according to World Health Organization 2023 standard criteria (WHO-2021) [50]. Semen samples were collected in sterile containers by masturbation after two to seven days of abstinence. After liquefaction, the quantitative and qualitative parameters of the sample (ejaculate volume, sperm count, sperm concentration, sperm motility, and sperm morphology) were evaluated. For sperm motility a CASA system (Microptic, Spain) was used with VCL settings as follow (VCL below 5 µm/S = non-progressive, VCL > 5 µm/S = progressive spermatozoa).

### Sperm DNA fragmentation assays

SDF was assessed via SCSA® and by the TUNEL assay using flow cytometry (FCM) in both cases. For the TUNEL assay, semen samples were washed twice with phosphate-buffered saline (PBS) and then fixed with 4% paraformaldehyde for 30 min. The washed semen samples were then permeabilized in 0.2% Triton X-100 (Merck, Darmstadt, Germany). The staining protocol was continued according to the instructions of the TUNEL assay supplier (Promega, Mannheim, Germany). TUNEL-positive cells were analyzed using a FACS-Calibur flow cytometer (BD Biosciences, San Jose, CA, USA). For each sample, at least 10,000 sperm cells were counted and the result was presented as the percentage of TUNEL-positive cells.

SCSA® was performed according to the protocol developed by Evenson (2013) [51, 52]. Briefly, two million fresh or flash frozen/thawed spermatozoa were suspended in a final volume of 1 ml of TNE buffer (50 mM Tris HCl pH 7.4, 100 mM NaCl, 0.1 mM EDTA; Merck, Darmstadt, Germany). After a 30 s treatment with an acid-detergent solution (0.08N HCl, 0.1% Triton X-100, pH 1.2), 6 µg/ml acridine orange (AO) staining solution (Sigma, St. Louis, USA) was added. The spermatozoa were then analyzed with a FACS-Calibur flow cytometer (BD Biosciences, San Jose, CA, USA). For each sample, at least 10,000 sperm cells were counted and the result was presented as the percentage of AO-positive sperm cells (green cells *versus* red/orange cells). The percentage of spermatozoa with AO staining above that of sperm with normal condensed chromatin, commonly referred to as the High DNA Stainability (HDS) population was also assessed [51, 52].

### Statistical analysis

All analyses were performed in the R environment (version 4.2.1 – R Core Team [2022]: R Foundation for Statistical Computing, Vienna, Austria). Descriptive statistics were applied to describe the main study parameters of minimum, maximum, and standard deviation from the mean. Because of the rejection of assumptions about normality of distribution and homogeneity of variance, comparisons of sperm parameters and DNA fragmentation between men of different age classes used a non-parametric Kruskall-Wallis test. When the Kruskall-Wallis test was significant (*p* < 0.05), pairwise Dunn tests were performed (Dunn post hoc test, Holm adjustment, p.adj < 0.05). For correlation analysis, the test of association between paired samples was based on Pearson’s product moment correlation coefficient.

## Results

The detailed characteristics of the semen samples in the cohort, as well as the specific number of samples for which data could be collected for each monitored parameter, are presented in Table 1. The age range of the cohort is 19 < years < 71 with a mean age of 37.5 ± 6.24 years. As it could be expected, the study population (mainly young male partners of infertile couples) is highly skewed, with a greater proportion of patients below the mean age than above the mean age (approximately 7000 versus 2500, respectively). This anomalous distribution could not be corrected by any statistical approach we attempted, precluding the use of parametric statistical analytical tests. Non-parametric statistical tests were therefore used. Rather than performing a linear analysis, we chose to perform an age-class analysis by examining the following male subgroups [20–29; N = 721]; [30–39; N = 5736]; [40–49; N = 2990]; [50–59; N = 241] (see Table 2). Older patients ([60–69; N = 41]; [70–79; N = 5]) were present in the study cohort, but their numbers were too small to be included in a valid and rigorous statistical analysis.

**Table 1:** Descriptive analysis of male age, semen parameters, and sperm DNA fragmentation from infertile men

Study parameters	N	Minimum	Maximum	Mean	Std. Deviation
Male age (year)	9735	19.00	71.00	37.5 ±	6.24
Semen volume (ml)	10,000	0.30	10.90	3.98	1.78
Sperm concentration (10^6^/ml)	10,000	0.10	494.55	60.18	44.3
Sperm count (10^6^/ejaculate)	10,000	0.10	2410.10	231.75	194.56
Total sperm motility (%)	10,000	0.00	100.00	44.67	22.25
Progressive motility “Fast + Slow” (%)	10,000	0.00	85.8	25.9	15.62
Non-progressive motility (%)	10,000	0.00	83.60	18.78	12.41
Immotile sperm (%)	10,000	0.00	100.00	55.32	22.25
Abnormal sperm morphology (%)	9977	61.00	100.00	94.92	4.58
SCSA High DNA stainability index (%HDS)	7353	1.00	34.00	8.5	4.14
DNA fragmentation index (%SCSA)	9774	5.00	75.00	18.56	7.52
DNA fragmentation (%TUNEL)	9961	1.00	60.00	10.3	6.16

**Table 2:** Comparison of sperm DNA fragmentation indexes between age classes of male patients

**Patient’s Age classes** *N* =	**20–29** *721*	**30–39** *5736*	**40–49** *2990*	**50–59** *241*	***P*** **-Value**
**SDF-TUNEL (%)**	8.39 ± 4.03a	9.47 ± 5.34b	11.1 ± 6.82c	13.9 ± 8.63d	< 2.2 e-16
**SDF-SCSA (%)**	16 ± 5.83a	17.7 ± 7.16b	19.9 ± 8.52c	23.8 ± 10.14d	< 2.2 e-16
**HDS SCSA (%)**	8.22 ± 4.31	8.54 ± 4.23	8.47 ± 4.06	8.02 ± 3.61	0.12
**Ratio** **SDF-SCSA/SDF-TUNEL**	1.91	1.87	1.79	1.71	-

The patients were divided into four groups according to age. Patients over age 59 years were not included in the analysis since this group contains only 46 individuals. Mean ± standard deviation of all parameters is indicated

For each variable, Kruskall-Wallis test was used to determine whether or not there is a statistically significant difference between the medians of the different groups of male age patients. The significance level was set to* P* < 0.05. When significant, a Dunn’s test was conducted (Holm adjustment) to determine which groups are different. Different lower-case letters indicate a significant difference among Patient’s groups

*HDS* High DNA Stainability, *SCSA* Sperm Chromatin Structure Assay, *SDF* Sperm DNA Fragmentation, *TUNEL* Terminal deoxynucleotidyl transferase dUTP Nick End Labeling

**Fig. 1:**
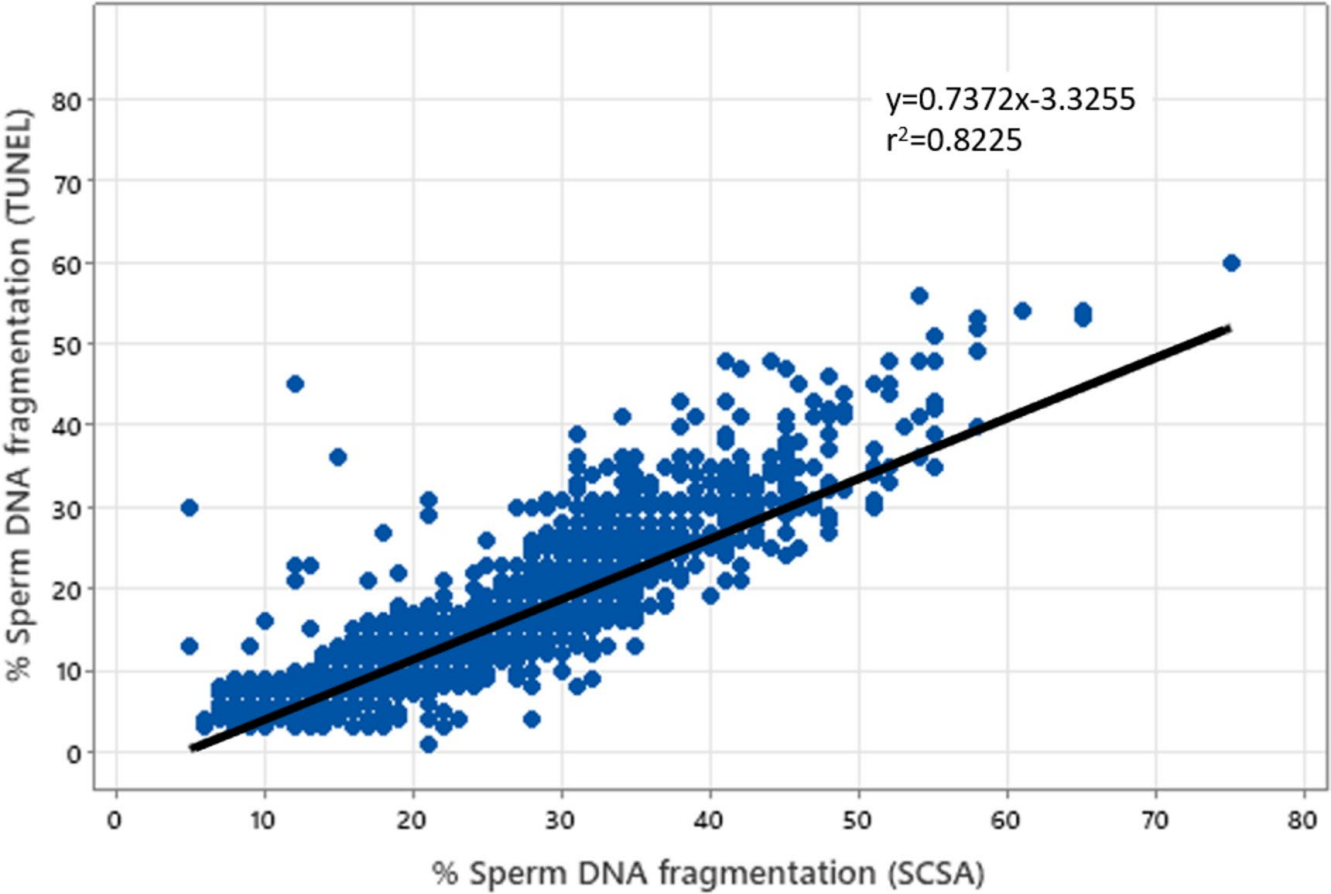
Correlation between sperm DNA fragmentation assessed by TUNEL staining, and sperm DNA fragmentation assessed by SCSA (r = 0.9; *p* < 0.001). Pearson's correlation coefficient was used for data analysis. The significance level was determined at *P* < 0.05. SCSA = Sperm Chromatin Structure Assay, TUNEL = Terminal deoxynucleotidyl transferase dUTP Nick End Labeling

**Fig. 2:**
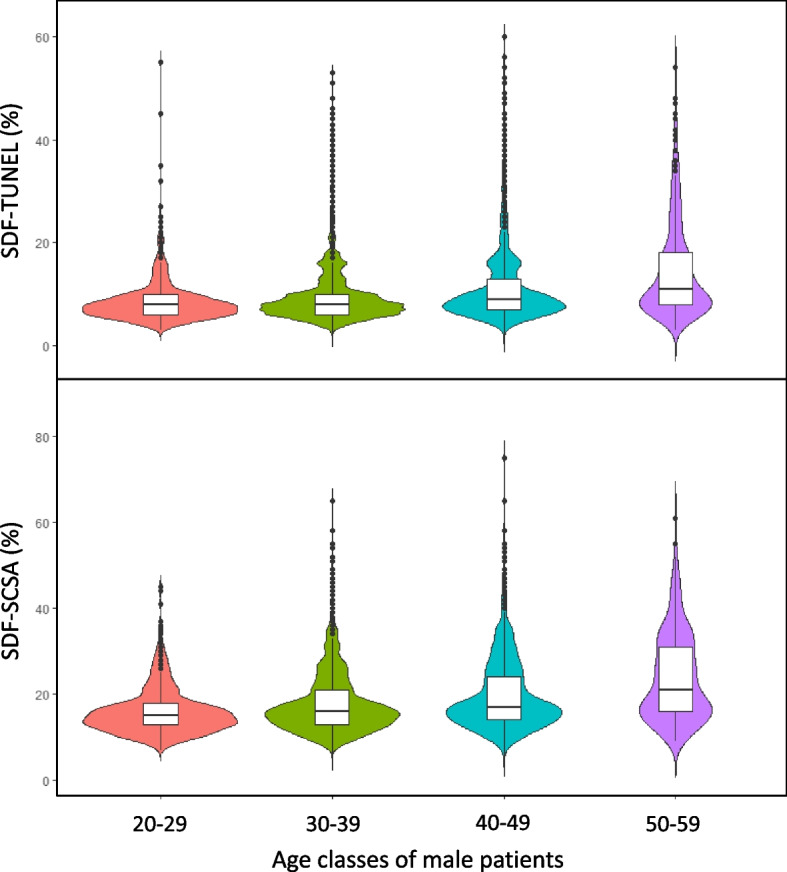
Violin plot graphical representation of SDF data by age group according to the TUNEL assay (top plots) or the SCSA assay (bottom plots)

Sperm DNA integrity was monitored by two concurrent assays, namely the SCSA® and the TUNEL assay. With both tests, it is clear that a linear SDF increase was observed with aging. Each age group analyzed was found to be statistically significantly different from each other (*p* = 2.2E-16; Table 2). A strong significant correlation (r = 0.9; *p* < 0.001) was observed between the two SDF assessments (Table 2 and Fig. 1). In comparing the two tests, we observed that the TUNEL test consistently yielded lower percentages for each age group compared with the SCSA® test (Table 2). SDF values obtained by the TUNEL assay were systematically lower but the relation was not linear when comparing age classes since the ratio SDF-SCSA/SDF-TUNEL tended to decrease upon aging, ranging from 1.91 for the youngest age class (20 to 29 years of age) going down to 1.71 for the oldest age class analyzed (50 to 59 years of age), (see Table 2). Extrapolating the SDF SCSA value to 30% considered as clinically relevant in predicting IVF failure, the SDF TUNEL equivalent would then be 18.79% (Fig. 1). The violin diagrams shown in Fig. 2 illustrate the distribution of data within each age group. They highlight the wide distribution of SDF in each age group, and the fact that ageing leads to a marked increase in the number of samples with higher levels of SDF.

Interestingly, the High DNA Stainability (HDS) parameter, which is the second parameter provided by the SCSA® FCM-assisted test, showed no significant difference for any age class.

**Fig. 3:**
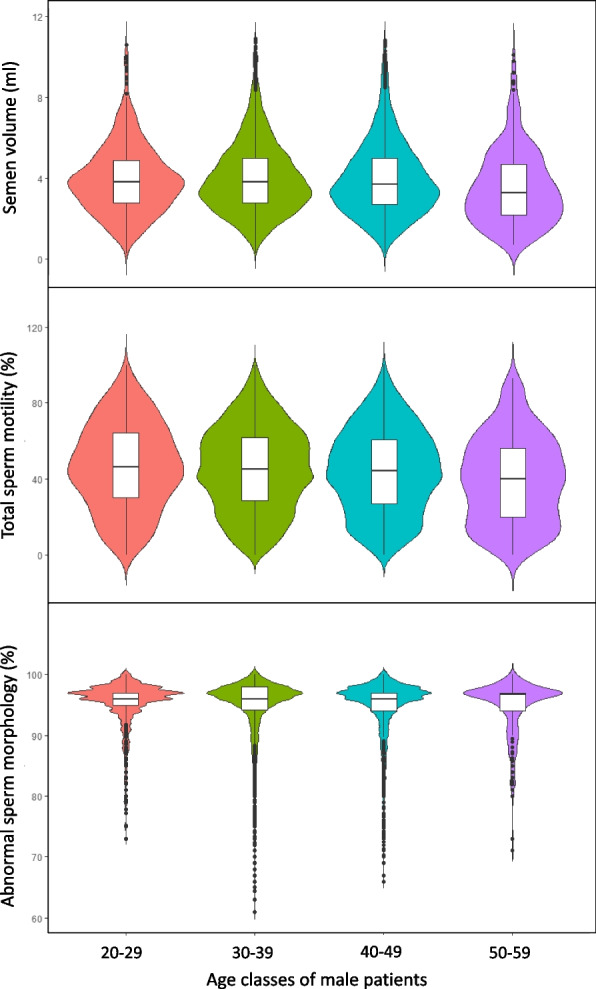
Violin plot graphical representation of data on sperm volume (top plots), total motility (middle plots) and sperm morphology (bottom plots) as a function of age class

Analyzing the influence of age on classical seminal/sperm parameters [50], we present in Table 3 (and illustrate in Fig. 3 via a violin-shaped graphic representation) that semen volume was found significantly different (*p* = 1.04E-6) and lower only in the last age group analyzed [50–59 years of age]. Similarly, total sperm motility was statistically different (*p* = 9.4E-6) only in the oldest age group analyzed compared to the other three younger age groups. Finally, sperm morphology was statistically different only in the oldest age group analyzed [50–59 years of age], although the *p* value obtained was less significant (*p* = 0.03). Sperm count and sperm concentration were not significantly different when the 4 age groups were compared.

**Table 3:** Comparison of age and classical semen parameters between groups of male patients

Patient’s Age classes	20–29	30–39	40–49	50–59	*P*-Value
**N samples**	720	5737	2891	342	-
**Male age (year)**	27.4 ± 1.65	34.8 ± 2.68	43.1 ± 2.64	52.5 ± 2.51	-
**Semen volume (ml)**	3.88 ± 1.63a	3.99 ± 1.7a	3.95 ± 1.77a	3.63 ± 1.77b	1.04 e-06
**Sperm concentration (10**^**6**^**/ml)**	60.4 ± 45.2	59.8 ± 41.6	62.8 ± 47.3	67.5 ± 49.4	0.11
**Sperm count (10**^**6**^**/ejaculate)**	230 ± 194	231 ± 182	239 ± 207	228 ± 186	0.47
**Total sperm motility (%)**	46.6 ± 22.7a	45.3 ± 22.1a	44.6 ± 22.5a	40.7 ± 22.4b	9.4 e-06
**Abnormal sperm morphology (%)**	94.5 ± 4.54ab	94.2 ± 5.06b	93.9 ± 5.37a	93.9 ± 4.85ab	0.03

The patients were divided into four groups according to age. Patients over age 59 years were not included in the analysis since this group contains only 46 individuals. Mean ± standard deviation of all parameters is indicated

For each variable, Kruskall-Wallis test was used to determine whether or not there is a statistically significant difference between the medians of the different groups of male age patients. The significance level was set to* P* < 0.05. When significant, a Dunn’s test was conducted (Holm adjustment) to determine which groups are different. Different lower-case letters indicate a significant difference among Patient’s groups

## Discussion

In the present study, we used a large cohort of male patients reaching nearly 10,000 semen samples. Semen samples were evaluated according to standard WHO procedures [50] for concentration, motility and morphology. In addition, each semen sample was evaluated for sperm DNA integrity using two of the most commonly used tests to assess SDF (i.e., SCSA® and the TUNEL test) as an indicator of sperm DNA/nuclear integrity. Patients were then classified into age groups and the mean values of the different monitored parameters were compared. We found that SDF increases steadily and significantly with age, regardless of the test used to determine it, either TUNEL or SCSA®. Our SCSA® data are in good agreement with two recent studies that also used large cohorts (over 10.000 semen samples each) and SCSA® to assess SDF during aging [46, 47]. Our analysis showed a good correlation between the SCSA® and TUNEL FCM-assisted assessment of SDF. This observation is consistent with previous reports that also showed good correlations between the two tests [53–60].

Interestingly, while TUNEL and SCSA® SDF values behave similarly during aging, TUNEL values are consistently lower than SCSA® SDF values. Although based on a very small cohort (n = 35), the SCSA® values reported in the Erenpreiss study were also roughly 2-times higher than the TUNEL values for the same sample, which agrees well with our observation [55]. The lower sensitivity of TUNEL in detecting SDF compared to SCSA® has been reported elsewhere [61, 62]. This is closely related to the different nature of the two tests [63] and to the peculiarity of spermatozoa. Indeed, human spermatozoa lack APE1 or XRCC1 activities, and consequently 3'-hydroxyl groups that in any other cell would result from DNA base repair activities [29]. This partly explains why the TUNEL sperm assay is rather insensitive, as it relies on the adduction of the terminal deoxynucleotidyl transferase (TdT) enzyme to the 3'-hydroxyl ends of double or single stranded DNA (DSB or SSB) breaks. In the SCSA® technique, sperm cells are incubated in suspension with a mild acid solution. The cells are then stained with acridine orange (AO), which fluoresces red when bound to single-stranded DNA and green when intercalated into the double helix [64]. Because AO is much smaller than TdT, it is assumed to involve many more cells with nuclear alteration, whether it is decondensation or/and the presence of DNA breaks. In this regard, it is generally accepted that the TUNEL assay detects existing breaks while the SCSA® assay detects existing and putative breaks [57, 65–69].

Previous studies in which the DNA Fragmentation index (DFI) was used to assess the correlation of SDF with age are available in the literature [70–73]. Roughly speaking, in these studies, DFI is twice as high in men over 45 years of age as in men under 25 years of age. The DFI indices that these studies report, however, are slightly higher (DFI of about 30% for men over 45 years of age) compared to what we have recorded in the present study. It is difficult to compare these studies with the present study because of the size of the cohorts, the likely different nature of the cohorts, and, most importantly, the lack of standardization to compare the measurement of DFI in one center versus another [74]. However, the general trend is similar with a steady increase in DFI during aging. This was confirmed by the conclusion of a meta-analysis based on 26 different studies cumulating just over 10,000 patients in which age was indeed associated with an increase in DFI [4].

In addition to DFI, it is interesting to note that in the cohort analyzed, the SCSA® HDS value was not associated with a significant change in the different age groups. This confirms our previous report suggesting that HDS is not a relevant discriminating parameter to monitor for assessing male fertility [75]. This suggests that overall, there is no significant change in sperm nuclear condensation/compaction during aging, as HDS is assumed to reflect the level of sperm nuclear protamination [51, 52]. Given that a high HDS value has been associated with poor embryo development and lower implantation rate [76, 77], events also associated with defective sperm cells during aging, it is interesting that impaired nuclear condensation does not appear to be the primary cause of defective spermatozoa associated with aging in our cohort. Given that we observed that sperm DFI increases significantly with aging, one could extrapolate that loss of sperm nuclear integrity with aging is more associated with direct DNA strand breaks than with lower nuclear compaction. This is consistent with the proposed explanation that the well-known mild pro-oxidant systemic context associated with aging tends to slightly increase sperm nuclear compaction during epididymal maturation, as it has been demonstrated in animal models [78–80]. This is also in agreement with the finding of the large (> 25,000 human semen samples) SCSA® cohort [47] that showed that the HDS parameter decreased slightly (ie. nuclear sperm condensation increased slightly) during aging. However, it should be mentioned that using chromomycin A3 (CMA3) staining, conflicting reports have shown a decrease in sperm DNA compaction with age in humans and animals [81, 82].

Regarding standard semen parameters, we report with the analysis of this large cohort that semen volume, total sperm motility and, abnormal sperm morphology are the only parameters that show a significant negative correlation with men's age. However, this is only true for the oldest age group analyzed [50–59 years of age]. In the other age groups and including the other parameters monitored (sperm concentration, sperm count), no significant difference was recorded between the age groups. In full agreement with our observations, Kidd et al. also reported a decrease in ejaculate volume, sperm normal morphology, and, motility, but not in sperm concentration, when comparing men of about 30 years of age with men of about 50 years of age [17]. Similarly, a large prospective study of 3,729 male partners [83] reported a significant decrease in sperm volume and motility with increasing paternal age. Several other retrospective studies have associated lower semen volume, lower progressive motility, and higher abnormal morphology in older men than in younger men [84–89]. In contradiction, Ereinpress et al. (2004) [55] reported that sperm concentration was negatively influenced by paternal age. Also, in contradiction to our observations, Hossain et al. (2012) [90] reported earlier that sperm count was negatively influenced by paternal age. Another study reported that with increasing male age, sperm concentration increased and that no difference could be observed in sperm motility and morphology [3]. These discrepancies are probably due to the characteristics of the cohorts studied (cohort size, healthy *vs* infertile men, sexual abstinence period, ethnicities, …) [31].

Because of the simultaneous evaluation of semen samples by 2 FCM-assisted SDF assays (SCSA® and TUNEL) each requiring at least 5 million cells, semen samples containing less than 10 million cells were excluded from the study. In addition, in this study, individuals were asked to provide semen after 2–7 days of abstinence, but the exact duration of abstinence was not recorded. This could impact the data, as has been recently demonstrated elsewhere [31]. These may be considered limitations of this study.

## Conclusions

In conclusion, the optimal reproductive age in humans has been studied for many years with a strong gender bias. Because men continually produce gametes as they age, whereas women have a fairly strictly defined gamete production window, much attention has been paid to female gametogenesis and to the rapid decline in oocyte performance as they age. The focus has also logically been on female gametes because of their major role in maintaining the early stages of embryo development, while spermatozoa have been neglected as a mere vehicle for the paternal DNA needed to re-establish diploidy through fertilization. These characteristics have long misled us into blaming females primarily for developmental failure and infertility. It is now quite clear that the male gamete takes its full share of responsibility for the developmental failures of the embryo as well as for the inheritance of defects in the offspring. In fact, due to their extreme cytodifferentiation, mammalian spermatozoa are more susceptible to DNA damage than oocytes. It is also now well established that the onus is on the oocyte to correct sperm DNA damage and that failure to do so can result in developmental arrest or the transmission of deleterious mutations inherited from the father to the offspring. A recent report showed that male aging is clearly associated with sperm DNA damage and reduced IVF/ICSI success rates [91].

In this context, it is clear that increasing attention should now be paid to the quality/integrity of sperm genetic material, although this is not yet fully translated into routine testing in ART techniques, despite recent awareness of international agencies (ASRM, ESHRE). The present work, performed on a large cohort, shows that sperm DNA fragmentation increases significantly with aging. It also shows that for each age group analyzed, the SDF range is quite wide, which in our opinion justifies evaluation regardless of the patient's age. It rather convincingly shows, for the first time on a large cohort, that the SCSA® and TUNEL tests assisted by FCM give concordant results, although the SCSA® appears to be more sensitive than the TUNEL test. Based on our data, we can roughly extrapolate that in our clinic, an FCM-assisted TUNEL DFI of 15% could be considered pathological as it could be translated into an SCSA DFI of approximately 30%. *In fine*, the age of the male partner must absolutely be factored into the equation when it comes to reproductive success and offspring health. In our opinion, given the wide range of SDF values in each age group, this should prompt us to monitor it to better characterize the male partner of an infertile couple and the associated risks.

## Data Availability

All data generated or analyzed in this study are provided.

## Abbreviations

AMP: Assistance Médicale à la Procréation
AO: Acridine Orange
APE1: Apurinic/apyrimidinic endonuclease 1
ART: Assisted Reproductive Technologies
ASRM: American Society for Reproductive Medicine
CASA: Computer-Assisted Spermatozoa Analysis
CMA3: Chromomycin A3
DFI: DNA Fragmentation Index
DNA: DesoxyriboNucleotidic Acid
EDTA: Ethylene Diamine Tetra-Acetate
ESHRE: European Society for Human Reproduction and Embryology
FACS: Fluorescence-Assisted Cell Sorting
FCM: Flow CytoMetry
HDS: High DNA Stainability
PBS: Phosphate Buffer Saline
SCSA: Sperm Chromatin Structure Assay
SDF: Sperm DNA Fragmentation
SSC: Sodium Citrate
TNE: Tris sodium (Na) EDTA
TUNEL: Terminal deoxynucleotidyl transferase dUTP Nick End Labeling
VCL: Curvilinear Velocity
WHO: World health Organization
XRXX1: X-Ray Repair Cross Complementing 1
